# Nanotechnology and Microtechnology in Drug Delivery Systems

**DOI:** 10.1177/1559325820907810

**Published:** 2020-04-15

**Authors:** Jingyao Sun, Zhaogang Yang, Lesheng Teng

**Affiliations:** 1 College of Mechanical and Electrical Engineering, Beijing University of Chemical Technology, Beijing, China; 2 Department of Radiation Oncology, University of Texas Southwestern Medical Center, Dallas, TX, USA; 3 Department of Biopharmaceutics, School of Life Sciences, Jilin University, Changchun, Jilin, China; 4 Key Laboratory for Molecular Enzymology and Engineering of Ministry of Education, School of Life Sciences, Jilin University, Changchun, Jilin, China

**Keywords:** nanotechnology, microtechnology, drug delivery systems, controlled drug release

## Abstract

A special issue of the journal *Dose-Response* entitled “Nanotechnology and Microtechnology in Drug Delivery Systems” is proposed. In pharmaceutical studies, new and existing drugs continue to be investigated for their poor specificity, solubility, therapeutic index, and immunogenicity. In order to solve these problems, drug delivery systems are essential for controlled drug release. It has been shown that the size and shape (nano- or micro-) of drug carriers can affect a drug’s circulation time, distribution, and cellular uptake. Hence, it is not surprising that nanotechnology and microtechnology have been explored as powerful tools for drug delivery in past decades. The main topics will be related to the technologies including microtechnology for the sustained release of drug, nanotechnology for the targeting delivery of drugs, new polymer materials nanotechnology, nanotechnology in drugs combination application, and so on.

The application of nanotechnology and microtechnology in drug delivery has changed the landscape of pharmaceutical and biotechnology industries in the 21st century. Nano and micro drug delivery systems can improve the therapeutic response by providing more stable blood drug levels than direct or sustained-release parenteral administration. Therefore, one of the development goals for drug delivery system is to enhance the drug efficiency with improved comfort, safety, and compliance for patients. These novel drug delivery systems may also make it possible to get improved efficacy with smaller quantities of drug.

In the past few decades, the latest advances in drug delivery system have made use of micro- and nanotechnologies that developed from the integrated circuit industry. Some of these technologies are making progress on the market. Some of these technologies have already been marketed and used for precise drug delivery. The requirements for new drug delivery technologies include higher personalization, automation, accuracy, and efficacy, together with less invasive and painful administration, and fewer side effects. Thus, the researches on new control release mechanism, the micromation and integration of biosensors, new materials and technologies for targeting drug delivery, and some other topics on nanotechnology and microtechnology in drug delivery systems are badly needed.

Microtechnology for drug delivery can be defined as the construction or assembly of delivery components on microscale to form a complete device or a key components of a larger delivery system. Microelectromechanical systems (MEMS) has become an emerging technology via the developments of miniaturization, multifunctional integration, and some other technologies to achieve the spatiotemporal controlled release of drugs for a wide range of targeting areas. Microneedle arrays are widely used in intraocular and transdermal drug delivery. Through the application of internal and external stimulation for intraocular, gastrointestinal, and subcutaneous drug delivery, it is now possible to achieve on-demand or self-regulation drug delivery using implantable systems. Besides the techniques of MEMS, the application of other traditional microfabrication technologies has also become alternative approach for the manufacturing of drug delivery systems. For example, the implantable microchips can be used for long-term treatment and complex dosing release based on individualized dosing regimen. Many other drug delivery systems including biocapsules, microparticles, microreservoirs, microfibers, and implantable pumps to enable access to diverse routes of administration were also invented and developed in recent years. These small size systems could realize new approaches for drug delivery with higher efficiency that impossible for conventional devices and needles. With the rapid development of computer science, the aforementioned microsize drug delivery systems would find their way from the desktop to the pharmacy in the foreseeable future.

In the past decades, nanotechnology for the targeting delivery of drugs has experienced rapid development and change. Among different kinds of emerging technologies, DNA nanotechnology provides a simple and powerful design technique for self-assembly of nanostructures, which has unique advantages and potential for the enhancement of drug targeting and the reduction of drug toxicity. Various sequence planning and optimization methods have been developed to design DNA nanostructures to improve the drug uptake efficiency of cells. These developments have implicated a bright future for nanomedicines and drug delivery techniques supported by DNA-based nanotechnology. The reasonable utilizations of new polymer materials nanotechnology and nanotechnology in drugs combination application are also becoming more popular because it can produce synergistic effect for the treatment of diseases through different mechanisms. The application drugs combination also can reduce individual drug-related toxicity and inhibit multidrug resistance.

This timely themed issue focuses on the application of new nanotechnology and microtechnology in drug delivery systems and methods. These recent developments offer unique opportunities to achieve the maximum therapeutic efficacy of drugs and meet the drug release profile required for a variety of clinical needs. The articles in this issue are reviewed by leading experts researching on the disciplines of physics, chemistry, material sciences, pharmaceutical sciences, medicine, and engineering. We are grateful to the experts who have contributed to this exciting issue and to the reviewers for their insights and suggestions.

